# Supplementary material for: Effects of Arylsulfatase B and Pembrolizumab in combination on progression of metastatic melanoma in the B16F10 syngeneic mouse model
Source: Front Oncol. 2026 Jun 1;16:1820206. doi: 10.3389/fonc.2026.1820206 (PMC13265294; doi:10.3389/fonc.2026.1820206)
Supplement: Supplementary Figure 1 — ARSB inhibits progression of subcutaneous B16F10 tumors and development in humanized mice with A375 tumors. (A) Treatment by rhARSB (0.2 mg/kg in ~40 µl saline) around the rim of B16F10 subcutaneous tumors in C57BL/6J mice was undertaken for 7 consecutive days in 7 female mice with measureable right flank melanomas. Previous work had treated with rhARSB preemptively on a fixed schedule, prior to the presence of measureable tumors. Initial procedures for tumor inoculation were similar to previous methods (5). Survival was compared to 8 untreated mice which were treated with similar volume of normal saline at similar time points. Tumor volume was reduced by ARSB treatment at 6 of 7 time points. Survival was increased in the treated mice, from mean of 26 days in the controls to 31 days in the treated mice (p=0.012, log-rank test). (B) Immune-deficient female mice NSG™ humanized mice (NOD.Cg-Prkdcscid Il2rgtm1WjlSzJ; strain #005557, Jackson Labs, Bar Harbor, ME) were engrafted with human PBMC and subsequently inoculated with 200,000 A375 human melanoma cells subcutaneously in the right flank. Treatment was rhARSB (0.2 mg/kg SQ at a concentration of 50 µg/ml saline) on days 2, 7, 14, 21, and 28 following tumor inoculation. Seven mice were treated with rhARSB, and 8 controls were treated with similar volume of saline on the same schedule. Mice were euthanized on day 31, when two of the untreated mice reached endpoint for tumor volume. All of the untreated mice had measureable tumors. In contrast, no tumors were palpable or visible in the treated mice. [file DataSheet1.pdf]

SF1. ARSB inhibits progression of subcutaneous B16F10 tumors and development in humanized mice with A375 tumors.

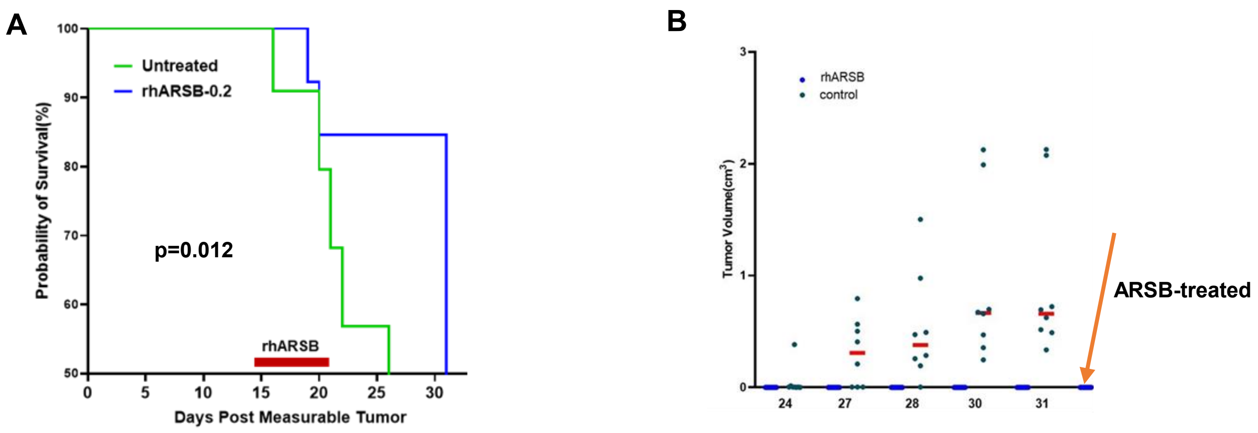

Supplementary Fig.1. ARSB inhibits progression of subcutaneous B16F10 tumors and development in humanized mice with A375 tumors.

A. Treatment by rhARSB (0.2 mg/kg in ~40 µl saline) around the rim of B16F10 subcutaneous tumors in C57BL/6J mice was undertaken for 7 consecutive days in 7 female mice with measureable right flank melanomas. Previous work had treated with rhARSB preemptively on a fixed schedule, prior to the presence of measureable tumors. Initial procedures for tumor inoculation were similar to previous methods [5]. Survival was compared to 8 untreated mice which were treated with similar volume of normal saline at similar time points. Tumor volume was reduced by ARSB treatment at 6 of 7 time points. Survival was increased in the treated mice, from mean of 26 days in the controls to 31 days in the treated mice (p=0.012, log-rank test).

B. Immune-deficient female mice NSG^{TM} humanized mice (NOD.Cg-*Prkdc*scid Il2rgtm1WjlSzJ; strain #005557, Jackson Labs, Bar Harbor, ME) were engrafted with human PBMC and subsequently inoculated with 200,000 A375 human melanoma cells subcutaneously in the right flank. Treatment was rhARSB (0.2 mg/kg SQ at a concentration of 50 µg/ml saline) on days 2, 7, 14, 21, and 28 following tumor inoculation. Seven mice were treated with rhARSB, and 8 controls were treated with similar volume of saline on the same schedule. Mice were euthanized on day 31, when two of the untreated mice reached endpoint for tumor volume. All of the untreated mice had measureable tumors. In contrast, no tumors were palpable or visible in the treated mice.

SF2. Modifiers of MMP expression in A375 cells

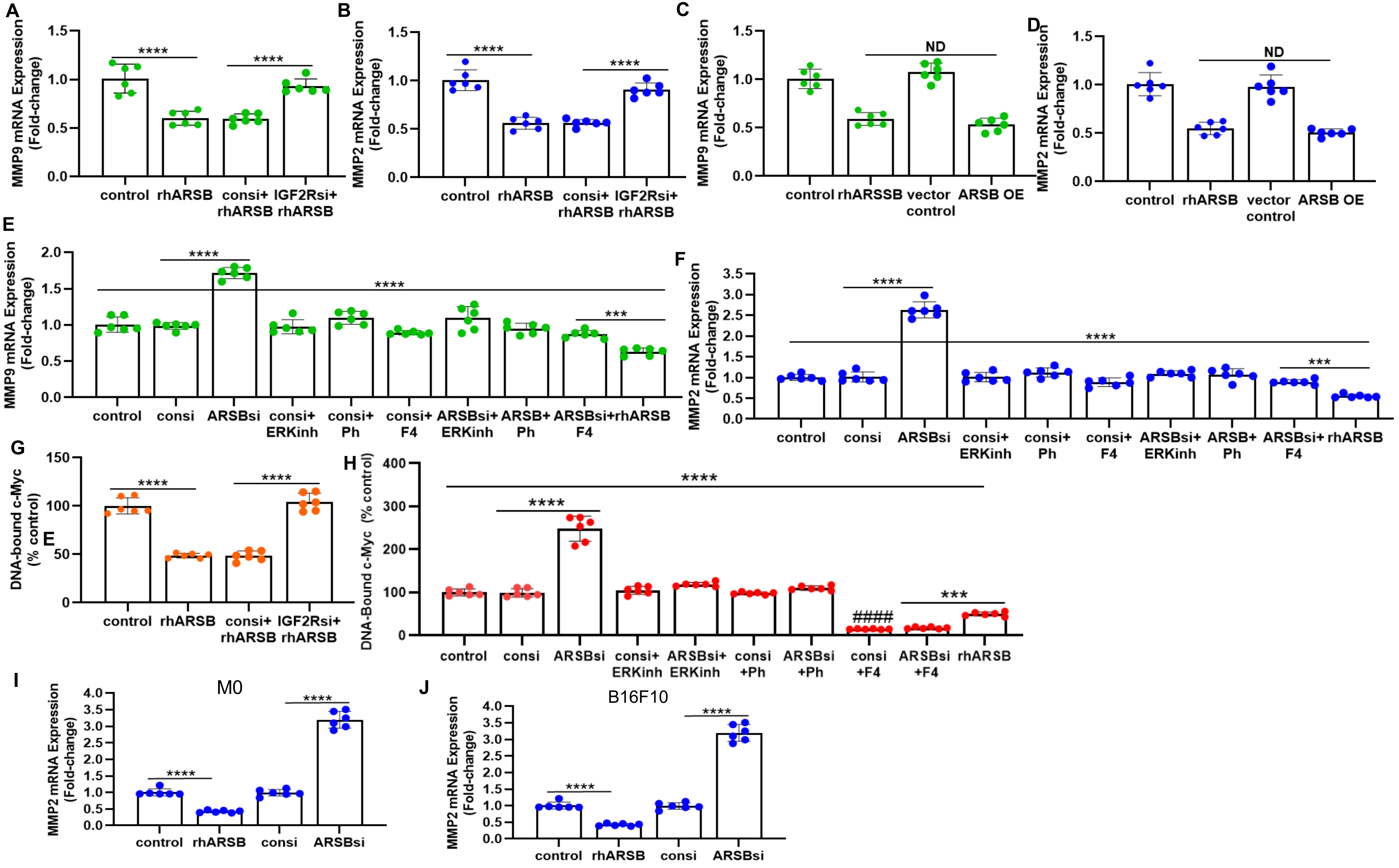

Supplementary Fig.2. ARSB-induced effects on MMP expression in A375 cells by effects on IGF2R and c-Myc.
A,B. In A375 cells, the inhibitory effect of rhARSB on MMP9 and MMP2 expression was blocked by knockdown of the IGF2R (insulin-like growth factor 2 receptor), the mannose 6-phosphate receptor which enables uptake of rhARSB [4,13].
C,D. Overexpression of ARSB by specific plasmid had a similar effect on MMP9 and MMP2 expression as treatment by rhARSB. Overexpression was performed using ARSB plasmid in pCMV6-XL4 vector (OriGene, Rockville, MD) and introduced in A375 cells by transient transfection using 2 µg of the plasmid and Lipofectamine^{TM} 2000 (Invitrogen, Thermo Fisher Scientific). Previously, controls included untransfected cells and cells transfected with empty vector control (OriGene). Media were exchanged after 6 h. Cells were incubated in a humidified, 37°C 5% CO_2 environment and harvested at 24 h after transfection. Effectiveness of transfection was determined by measurements of ARSB activity before and after transfection. ARSB activity was measured as previously reported [3,6].
E,F. Increases in MMP9 and MMP2 mRNA were inhibited by ERK activation inhibitor peptide 1, cell-permeable (ERKinh), p38-MAPK inhibitor (Ph), and c-Myc/Max inhibitor (F4; thiazolidinone) which blocks c-Myc-Max dimerization. Prior experiments in subcutaneous B16F10 melanomas and in cultured melanoma cells showed that ARSB affected the expression of MMP2 and MMP9 through SHP-2-mediated effects on phospho-ERK1/2 and subsequent effects on DNA binding of c-Myc [5,6]. Similar effects are shown in the A375 cells.
G. DNA-bound c-Myc was inhibited by treatment with rhARSB in A375 cells. Knockdown of IGF2R, which is required for cellular effects of exogenous rhARSB, inhibited this response [4,13].
H.. ERK activation inhibitor peptide 1, cell-permeable (ERKinh), p38-MAPK inhibitor (Ph), and c-Myc/Max inhibitor (F4) blocked the increase in nuclear-bound c-Myc. ARSB siRNA increased DNA-bound c-Myc, and this increase was inhibited by ERK inhibitor, p38-MAPK inhibitor, and c-Myc/Max inhibitor (cell-permeable thiazolidinone, labelled F4), which completely suppressed the effect of ARSB silencing on DNA-bound c-Myc.
H. ARSB knockdown increased MMP2 expression in normal human melanocytes. The normal melanocytes were grown and treated as previously described [6,13]. Primary normal melanocytes were cultured in Airway Epithelial Cell Basal Medium (ATCC, Manassas, VA) with melanocyte growth kit (ATCC) and maintained at 37°C in a humidified, 5% CO2 environment with media exchange every 3 days. Confluent cells were harvested by trypsin for primary cells (ATCC) and sub-cultured.
I. Consistent with other findings, rhARSB significantly reduced mRNA expression of MMP2 and MMP9 (not shown) and ARSB siRNA significantly increased MMP2 and MMP9 (not shown) expression in B16F10 mouse melanoma cells. There was greater increase of MMP2 than MMP9 by ARSB knockdown. B16F10 mouse melanoma cells were purchased (ATCC CRL-6475) and cultured in DMEM supplemented with 10% FBS and 1% penicillin-streptomycin. Cells were screened for pathogens by IDEXX BioAnalytics (Columbia, MO). Cells were maintained at 37°C in a humidified, 5% CO2 environment with media exchange every 2 days, and confluent cells in T-25 flasks were harvested by EDTA-trypsin, and sub-cultured.

SF3. Cytokine expression following ARSB siRNA and rhARSB in A375 cells.

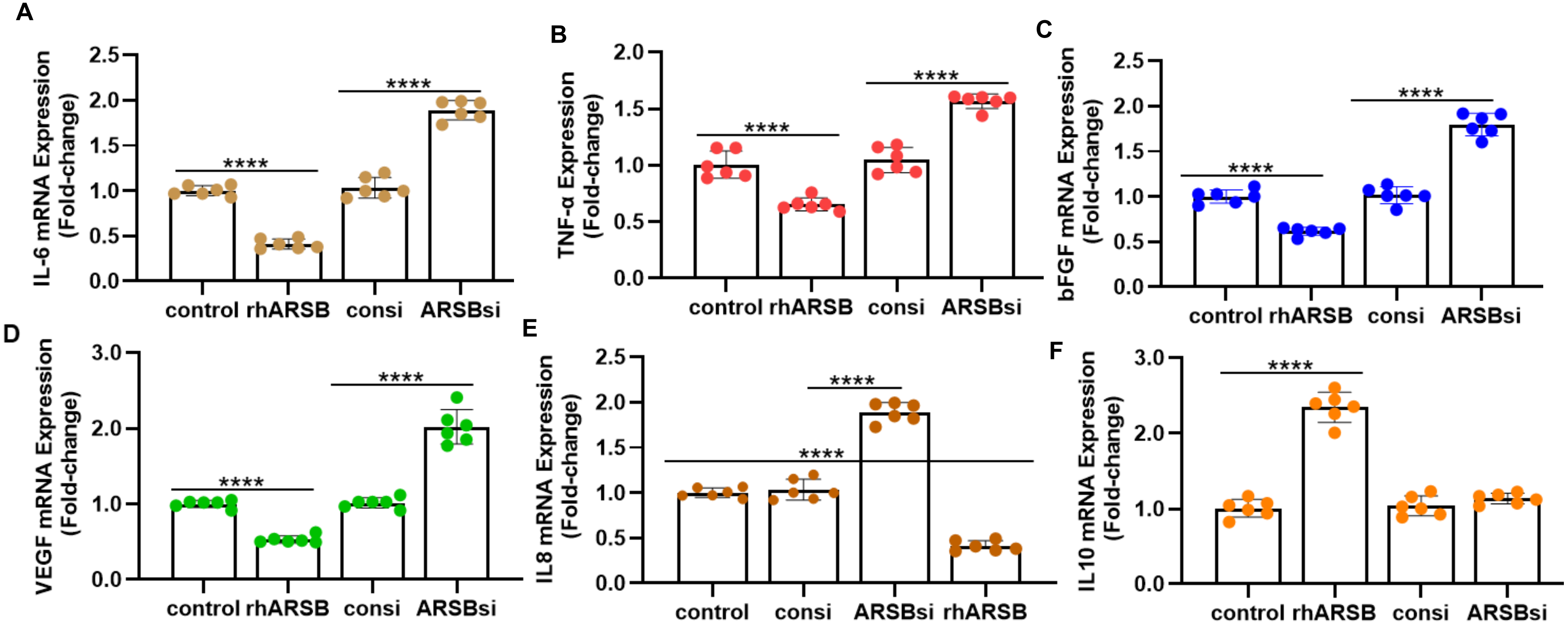

Supplementary Fig.3. Cytokines/Chemokines following ARSB siRNA and rhARSB in A375 cells.
A,B,C,D,E. mRNA expression data show inverse effects of rhARSB (declines) and ARSB siRNA (increases) on IL-6, TNF-α, bFGF, VEGF, and IL-8. These results are similar to findings from cytokine array.
F. IL-10 is increased by rhARSB and unaffected by ARSB siRNA.

SF4. Binding of IL-8 to C4S

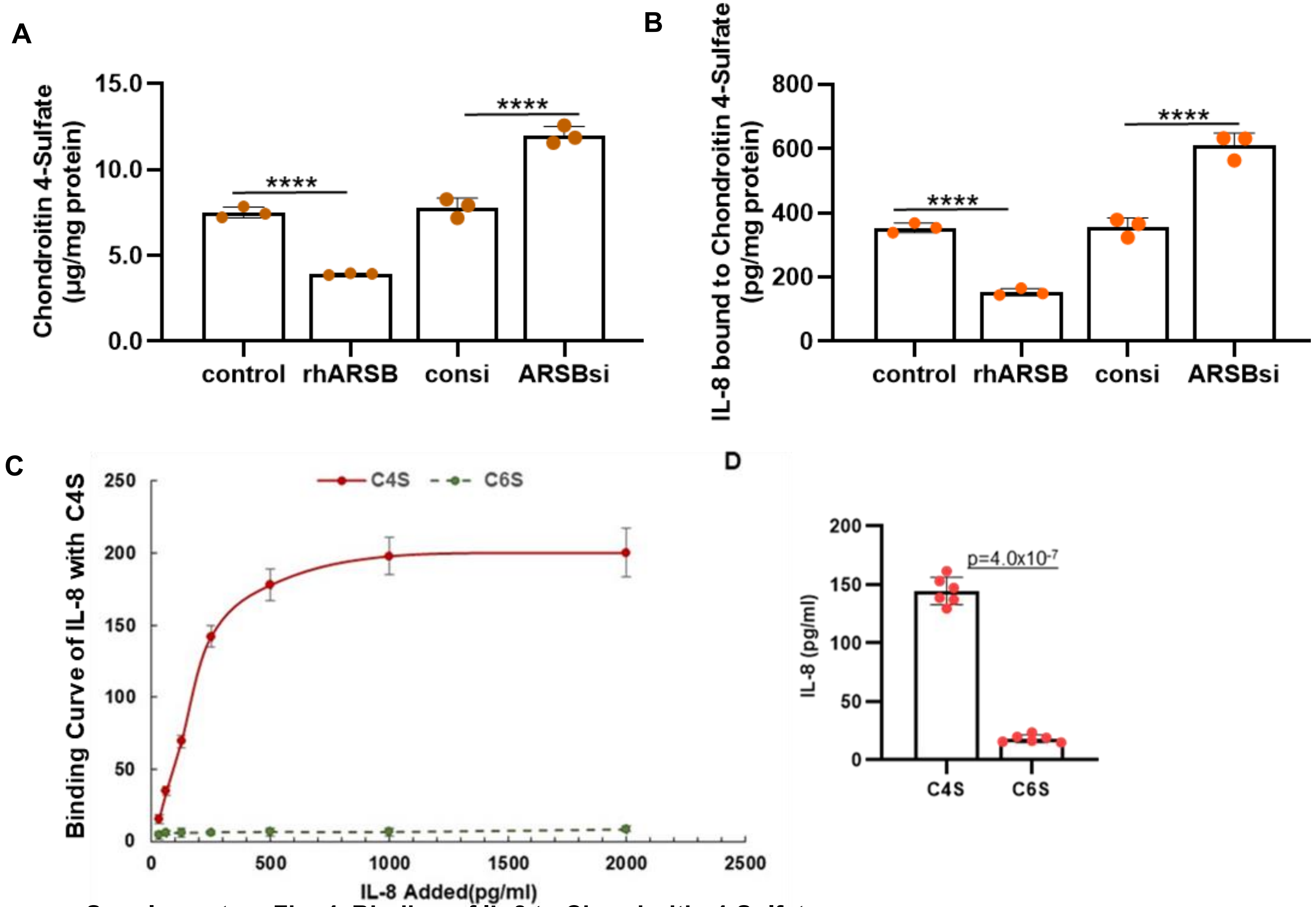

Supplementary Fig. 4. Binding of IL-8 to Chondroitin 4-Sulfate

A. Chondroitin 4-sulfate content in A375 human melanoma cells was reduced by rhARSB (1 ng/ml x 24h) and increased when ARSB was silenced by siRNA.

B. When rh IL-8 was added to wells of a 24-well plate coated with A375 cells, the amount of bound IL-8 declined when cells were treated with rhARSB and increased when ARSB was silenced, indicating that IL-8 binds more with more sulfated C4S present when ARSB activity is reduced. IL-8 in the cell extracts was measured by ELISA IL-8 (DY208, R&D Systems).

C. In an *in vitro* binding experiment, wells of a 24-well plate were coated with either C4S or C6S antibody (5 µg/ml) and C4S or C6S (100 µl; 2.5 µg/ml) was added. Subsequently, rhIL-8 at varying concentrations was added, biotinylated IL-8 antibody was added, and the extent of IL-8 binding detected by ELISA, as detailed in the Methods. Maximum binding of IL-8 with C4S was ~200 pg/ml. Virtually no IL-8 bound to chondroitin 6-sulfate.

D. When 250 pg/ml of IL-8 was added to wells coated with either C4S or C6S, 144.6 ± 11.7 pg/ml of IL-8 bound to C4S and 18.3 ± 3.2 pg/ml of IL-8 bound to C6S.

SF5. Binding of mouse PD-1 with Pembrolizumab

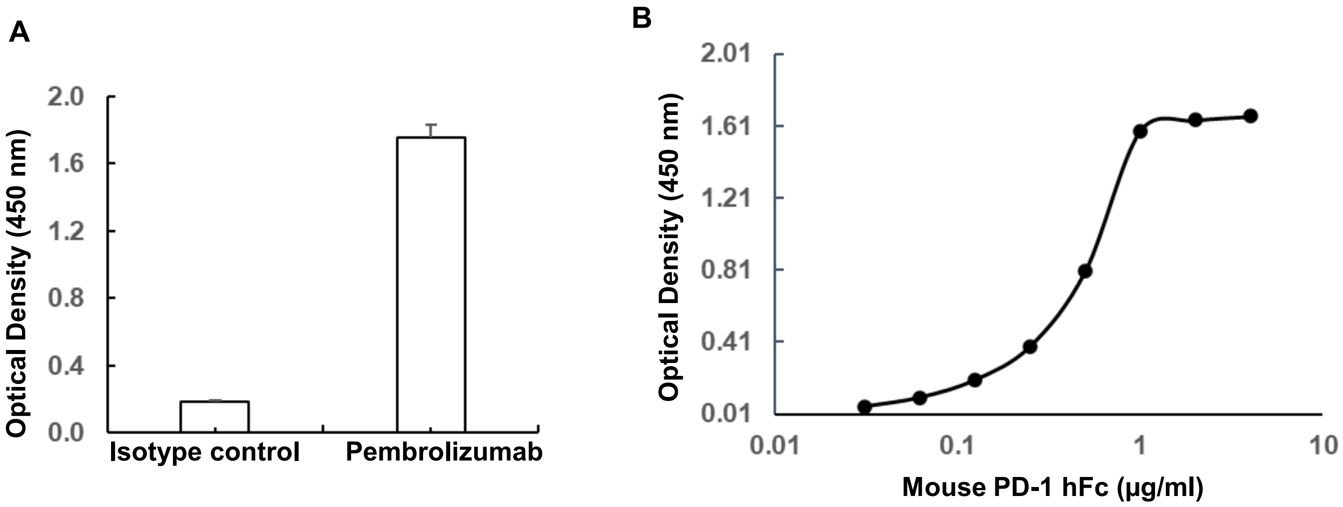

Supplementary Fig. 5. Binding of mouse PD-1 with Pembrolizumab
A. IGG4-kappa isotype control binding to mouse recombinant human Fc-tagged PD-1 was minimal. In contrast to binding to Pembrolizumab in coated wells.
B. Binding to Pembrolizumab increased with increasing concentration of mouse PD-1, peaking at ~1.61 µg/ml.
